# Comparative Transcriptome Profiling of a Resistant vs. Susceptible Tomato (*Solanum lycopersicum*) Cultivar in Response to Infection by Tomato Yellow Leaf Curl Virus

**DOI:** 10.1371/journal.pone.0080816

**Published:** 2013-11-18

**Authors:** Tianzi Chen, Yuanda Lv, Tongming Zhao, Nan Li, Yuwen Yang, Wengui Yu, Xin He, Tingli Liu, Baolong Zhang

**Affiliations:** Provincial key laboratory of agrobiology, Jiangsu Academy of Agricultural Sciences, Nanjing, China; Institute of Infectious Disease and Molecular Medicine, South Africa

## Abstract

Tomato yellow leaf curl virus (TYLCV) threatens tomato production worldwide by causing leaf yellowing, leaf curling, plant stunting and flower abscission. The current understanding of the host plant defense response to this virus is very limited. Using whole transcriptome sequencing, we analyzed the differential gene expression in response to TYLCV infection in the TYLCV-resistant tomato breeding line CLN2777A (R) and TYLCV-susceptible tomato breeding line TMXA48-4-0 (S). The mixed inoculated samples from 3, 5 and 7 day post inoculation (dpi) were compared to non-inoculated samples at 0 dpi. Of the total of 34831 mapped transcripts, 209 and 809 genes were differentially expressed in the R and S tomato line, respectively. The proportion of up-regulated differentially expressed genes (DEGs) in the R tomato line (58.37%) was higher than that in the S line (9.17%). Gene ontology (GO) analyses revealed that similar GO terms existed in both DEGs of R and S lines; however, some sets of defense related genes and their expression levels were not similar between the two tomato lines. Genes encoding for WRKY transcriptional factors, R genes, protein kinases and receptor (-like) kinases which were identified as down-regulated DEGs in the S line were up-regulated or not differentially expressed in the R line. The up-regulated DEGs in the R tomato line revealed the defense response of tomato to TYLCV infection was characterized by the induction and regulation of a series of genes involved in cell wall reorganization, transcriptional regulation, defense response, ubiquitination, metabolite synthesis and so on. The present study provides insights into various reactions underlining the successful establishment of resistance to TYLCV in the R tomato line, and helps in the identification of important defense-related genes in tomato for TYLCV disease management.

## Introduction

Tomato (*Solanum lycopersicum*) is an economically important vegetable, with 46.87% increase in total world production from 108 million tons in 2001 to 159 million tons in 2011 (http://faostat.fao.org/). China is a leading tomato producing industry and its production has doubled from 24 million tons between 2001 and 2011 (http://faostat.fao.org/). However, the increasing international travel and trade of plant materials as well as the changing climate have increased the incidence of tomato yellow leaf curl virus (TYLCV), which has caused serious damage to tomato crops [1,2]. TYLCV, which is transmitted by the whitefly *Bemisia tabaci* Gennadius (*Hemiptera*:*Aleyrodidae*) in a persistent and circulative manner, has recently spread to diverse geographic areas worldwide from its origin in the Middle East [3,4]. The symptoms of TYLCV infection in young plants include stunted growth, upward curling of leaf margins, marked reduction in leaf size, mottling and yellowing of young leaves, and flower abscission, leading to severe yield loss [1,4]. 

Since whitefly populations are difficult to control and a narrow genetic base of tomatoes is available, a major economic control strategy for this pathogen is to identify genetic resistance and transform the resistance genes into tomato [1,4–6]. Currently five major loci resistant to TYLCV (*Ty-1*, *Ty-2*, *Ty-3*, *Ty-4* and *Ty-5*) have been introgressed from different wild tomato relatives into tomatoes. Among these, *Ty-1* [7], *Ty-3* [8] and *Ty-4* [9] derived from different *S. chilense* accessions, *Ty-2* [10] originated from *S. habrochaites*, and *Ty-5* [11] was identified in *S. peruvianum*. Recently, *Ty-1* and *Ty-3* were found to be allelic and had been cloned [12,13]. Both *Ty-1* and *Ty-3* code for a RNA-dependent RNA polymerase (RDR) belonging to the RDRɤ type, which has an atypical DFDGD motif in the catalytic domain and may be involved in RNA silencing [12]. However, the resistance mechanism of these genes in TYLCV resistant tomatoes remains unclear. 

With a TYLCV resistant inbred line and a TYLCV susceptible inbred line from the same breeding program using *Solanum habrochaites* as the source of resistance, Gorovits [14] et al found that upon whitefly-mediated inoculation of TYLCV, the resistant lines revealed a less pronounced decline in the abundance of mitogen-activated protein kinases (MAPK), cellular heat shock proteins and chloroplast protease FtsH, and a less pronounced increase in the activities of the pathogenesis-related proteins, β-1,3-glucanase and peroxidase, than in the susceptible line. Comparison of protein profiles and metabolites patterns in TYLCV infected resistant and susceptible lines further revealed that higher levels of reactive oxygen species compounds, anti-oxidative proteins, pathogenesis-related and wound-induced proteins in the susceptible plants [15]. However, the sources of carbon and nitrogen were less affected and the host defense mechanisms were also much less activated by TYLCV in the resistant than in the susceptible plants [15]. The maintenance of medium-sized TYLCV coat protein (CP) aggregates was found to be associated with resistance, while larger aggregates were a characteristic of susceptibility in tomato, suggesting that the sequestering of virus CP into medium-sized aggregates and hindering the formation of large insoluble aggregates containing infectious particles is a part of the response of resistant plants to TYLCV [16]. Recently, screening cDNA libraries from resistant and susceptible lines before and after TYLCV inoculation resulted in the identification of 69 genes that were preferentially expressed in the resistant line [17]. Twenty-five preferentially expressed genes were tested and five genes, which respectively encode permease I-like protein, hexose transporter (LeHT1), lipocalin-like protein (SlVRSLip), chlorophyll a-b bingding protein 7 and thioredoxin peroxidase, were found to cause the collapse of resistance upon tobacco rattle virus-induced gene silencing [17–20]. The co-silencing of SlVRSLip, LeHT1 and permease curbed the growth of resistant plants upon infection and proliferation of the virus [20]. Moreover, the expression of SlVRSLip was inhibited in resistant plants in which LeHT1 was silenced, whereas the expression of LeHT1 was not inhibited in SlVRSLip silenced resistant plants, suggesting that SlVRSLip is downstream of LeHT1 [20]. These results of gene network and cellular response to TYLCV are preliminary and further investigations about tomato resistance to TYLCV are desirable.

The recently sequenced genomes of tomato and its wild relative *Solanum pimpinellifolium* [21] have provided great insights into the genetic and genomic based inquiries in tomato, and have helped in the identification of important genes in the family *Solanaceae*. This has accelerated the improvement in the worldwide tomato production to better combat the biotic and abiotic stresses decrease productivity in this plant. Next-generation RNA sequencing (RNA-seq), which has a greater dynamic range, detects both coding and non-coding RNAs [22,23], and is a powerful tool to detect differential gene expression [24,25]. RNA-seq technology has revealed several differentially expressed genes in many plants in response to pathogen attack, which in turn have helped in understanding the mechanism of host resistance and the complex nature of plant-microbe interactions [26–32]. Some tomato transcriptome data which focused on fruit biology, nematode resistance, plant hormones regulation and others but not on TYLCV defense are already available in tomato [33–38]. In this study, RNA-seq was used to compare transcriptional changes in a TYLCV-resistant tomato breeding line CLN2777A (R) and a TYLCV-susceptible tomato breeding line TMXA48-4-0 (S) in response to TYLCV infection. There was a higher proportion of up-regulated differentially expressed genes in the tomato R line than that in the S line. Some sets of defense related genes encoding for WRKY transcriptional factors, R genes, protein kinases and receptor (-like) kinases which exhibited a dramatic down-regulation in the S line were up-regulated or not differentially expressed in the R line. Further analysis of the up-regulated differentially expressed genes in the R line revealed at least 40 annotated genes associated with plant defense response at different levels ranging from the regulation of transcription factors to the activation of defense genes and to the post-translational modification of proteins that participate in the defense response to pathogen infection.

## Materials and Methods

### Plant material and TYLCV inoculation

Tomato plantlets from a TYLCV-resistant breeding line CLN2777A (R), which was derived from the H24 × CL5915 and carries the *Ty-2* locus [39], and a TYLCV-susceptible breeding line TMXA48-4-0 (S) were grown in insect-proof cages under 26°C and 16 h light/8 h dark conditions. Whiteflies viruliferous for the TYLCV-IL strain [40] were propagated and maintained with tomato plants in insect-proof greenhouse. Tomato plants at the two-leaf stage were inoculated with viruliferous whiteflies by exposing the plants to these flies in insect-proof cages for 3 days, which were then treated with an insecticidal imidacloprid to kill the whiteflies [12].

### Library preparation and sequencing

Leaf samples were collected at 0, 3, 5 and 7 days post inoculation (dpi) and frozen immediately in liquid nitrogen before use. For library preparation, leaf samples collected at 3, 5 and 7 dpi were pooled together for RNA isolation and made as a mixed infection library following the preparation strategy for the pooled cDNA library [17], which could represent gene expression at the early stages of infection; while leaves before TYLCV inoculation (0 dpi) served as a non-infection library. Total RNA was isolated using the RNAout kit (Tianze, Beijing, China) and treated with RNase free DNaseI (Ambion, Austin, USA) according to the manufacturer's guidelines. RNA concentration and quality were determined using the Agilent 2100 Bioanalyzer (Agilent Technologies, Santa Clara, USA). The mRNA was recovered from total RNA with the Micropoly(A) Purist^TM^ mRNA purification kit (Ambion, Austin, USA) and was quantified by a spectrophotometer NanoDrop (NanoDrop Technologies, Wilmington, USA). The construction of cDNA library was according to the described procedure [41]. Briefly, a GsuI-oligo dT primer was used to prime first-strand cDNA synthesis from 10 μg of mRNA, using 1000 units of Superscript II reverse transcriptase (Invitrogen, Carlsbad, USA). The diol group of the 5’CAP structure of mRNA was then oxidized by NaIO4 (Sigma, St. Louis, USA), followed by biotinylation with biotin hydrazide (Vector Laboratories, Burlingame, USA). After RNaseONE digestion, Dynal M280 streptavidin Dynabeads (Invitrogen, Shanghai, China) were used to select biotinylated RNA/cDNA. The first-strand cDNA was then released by alkaline hydrolysis, and two 5' adaptors (N5 and N6 adaptors) were ligated to the 5'-end of the first-strand cDNA. Double-stranded cDNA was synthesized by primer extension using Ex-Taq polymerase (TaKaRa, Dalian, China). The polyA tail and 5' adaptor were removed by GsuI digestion, and cDNA size fractionation was performed using a cDNA size fractionation column (Invitrogen, Shanghai, China). Each cDNA fraction larger than 800 bp was sonicated to the range of 300-500 bp. The purified cDNAs were used to construct libraries with Illumina's TruSeq^TM^ DNA Sample prep kit-Set A and were loaded onto an Illumina cBot for cluster generation with Illumina's TruSeq PE Cluster Kit as per the manufacturer's recommended protocols, followed by sequencing on the HiSeq 2000 sequencer. The raw sequencing data have been submitted to the NCBI Sequence Read Archive under accession number SRP028618.

### Processing of mRNA Sequence data

The raw RNA-Seq reads were preprocessed by FastQC (http://www.bioinformatics.bbsrc.ac.uk/projects/fastqc/) to remove the low-quality reads, and then mapped to the tomato genome [21] using a spliced read mapper Tophat [25] version 2.0 (http://tophat.cbcb.umd.edu). Transcript abundance and differential gene expression were calculated with the program Cufflinks [25] (http://cufflinks.cbcb.umd.edu/). Gene expression levels were normalized with fragments per kilobase of exon per million mapped reads (FPKM) values. The false discovery rate (FDR) was used to determine the threshold of P value in multiple test and analysis. In this study, genes were considered to be differentially expressed only when their absolute value of log_2_ fold change was >1 and FDR was <0.05. Functional annotation of differentially expressed genes was analyzed by Blast2go software (http://www.blast2go.org) with the default parameters. The major GO categories to which the differentially expressed genes belong were determined after the genes were subject to BLAST, mapping and annotation.

### Validation of differentially expressed genes by quantitative RT-PCR

Five genes which were associated with pathogen resistance in plants were selected and subjected to quantitative RT-PCR validation. Primers for quantitative RT-PCR were designed using Primer5 software and the primer specificity was evaluated by blasting primer sequences against the NCBI database. PCR amplifications were performed in a real-time thermal cycler qTOWER 2.0/2.2 (Analytik jena, Germany) with 25 μl of final volumes containing 2.5 μl of cDNA, 0.5 μl each primer (10 μM), 9 μl of sterile water, and 12.5 μl (2×) SYBR Premix ExTaqTM II Kit (TaKaRa). The conditions for amplification were as follows: 5 min denaturation at 95 °C followed by 40 cycles of 95 °C for 10 s, 60 °C for 20 s, and 72 °C for 10 s. The expression levels of selected genes were normalized to α-Tubulin (Solyc04g077020.2) expression. Relative gene expression was calculated using the 2^-ΔΔCT^ method. Three biological replicates for each of the selected genes were performed.

### Validation of candidate genes with virus-induced gene silencing (VIGS)

The tobacco rattle virus (TRV) mediated VIGS system [42] was used to silence a NBS-LRR resistance gene (Solyc05g009760.1). pTRV-containing *Agrobacterium* EHA105 was cultured in liquid LB medium with 50 mg·l^-1^ kanamycin and 25 mg·l^-1^ rifampin overnight at 28 °C. *Agrobacterium* cells were harvested and resuspended in infiltration media (10 mM MgCl_2_, 10 mM MES, 200 mM acetosyringone) to an O.D.value of 2.0 and cultured at room temperature for 4 h. For agroinfiltration, an equal volume of *Agrobacteria* containing of pTRV1 or pTRV2-(NBS-LRR) was mixed and infiltrated into the cotyledons of tomato seedlings at the cotyledon stage with 1 ml syringe. The agroinfiltration of pTRV1 with pTRV2-PDS and pTRV1 with empty pTRV2 served as positive control and negative control, respectively. All the tomato plants were grown in pots at 25 °C in a growth chamber under 16 h /8 h photoperiod.

Seven days after agroinfiltration, the plants were inoculated with TYLCV for a 3-day inoculation period as described above. One month after agroinfiltration, new emerging leaves from the TYLCV infected plants were used to extract RNA and DNA, which was subsequently used to determine the expression level of target gene (Solyc05g009760.1) and the accumulation of TYLCV DNA in the VIGS-treated plants by quantitative RT-PCR, respectively. The conditions and parameters of quantitative RT-PCR were the same as above. The primers used to amplify target gene were 5′-CTTTGCGGGTTCGTTCATCTTAT-3′and 5′-CGTTTATGTCCACATGCCTCAAC-3′, and the primers for accumulation detection of TYLCV DNA were 5′-GCCYATRTAYAGRAAGCCMAG-3′ and 5′-GANGSATGHGTRCADGCCATATA-3′. α-Tubulin (Solyc04g077020.2) was used as an internal reference.

## Results

### Differentially Expressed Genes in Response to TYLCV Infection

In order to detect gene expression at the early stages of infection, RNA isolated from the leaves of TYLCV-resistant tomato breeding line CLN2777A (R) and TYLCV-susceptible tomato breeding line TMXA48-4-0 (S), before (0 dpi) and after (3, 5 and 7 dpi leaves pool together for RNA isolation and library construction according to [17]) TYLCV inoculation with viruliferous whiteflies, was subjected to RNA-seq. The raw HiSeq reads were first filtered to discard the reads with average Phred quality score less than 20 and were further checked for sequence contaminants with the FastQC. About 8~18 million ~100bp clean pair end reads were obtained from each of the four libraries (Table 1), and these raw data have been deposited in the NCBI Sequence Read Archive (accession number SRP028618). The resulted ~100bp clean pair end reads were then mapped with TopHat to the tomato genome sequence [21], resulting in a total of 34831 transcripts (Table S1). Of which, 33446 (96.02%) transcripts corresponded to the predicted protein-coding genes in the tomato genome, and 1386 (3.98%) transcripts were novel transcripts which cannot link to predicted genes in tomato, suggesting the presence of uncharacterized transcriptionally active regions. 

**Table 1:** Numbers of aligned and mapped reads.

**Samples**	**Total filtered pair-end reads**	**Total mapped reads (%)**	**Uniquely mapped reads (%)**
**S before infection**	2×8359591	10534550 (63.01)	9250445 (55.33)
**S after infection**	2×16238068	24722213 (76.12)	22930427 (70.61)
**R before infection**	2×12423571	19432210 (78.21)	18269401 (73.53)
**R after infection**	2×18267613	28549270 (78.14)	26457199 (72.42)

Reads with average Phred quality score less than 20 and reads with bases less than 20bp are filtered out.

The expression levels of mapped genes were normalized with a value of FPKM (fragments per kilobase of exon per million fragments mapped). To confirm the quality of RNA-seq, fourteen highest-ranking housekeeping control genes in tomato leaves coding for GAPDH, phosphoglycerate kinase, catalase, Cys protease, tubulin, ubiquitin, actin, DnaJ chaperone and translation initiation factor 5A [43] were selected to evaluate gene expression. Based on pair-wise comparisons of samples before and after TYLCV inoculation, none of these reference genes were significantly differentially expressed in the R or S line (Table 2), suggesting that the sequences obtained and the transcript expression levels were qualified for further transcriptome analysis. 

**Table 2:** Expression levels of housekeeping control genes in tomato leaves.

**Housekeeping Gene^a^**	**TC Accession**	**Gene Id**	**R log_2_ fold change**	**FDR**	**Significant**	**S log_2_ fold change**	**FDR**	**Significant**
GAPDH	TC123860	Solyc04g009030.2	0.34	1.00	no	-0.58	1.00	no
GAPDH	TC124167	Solyc12g094640.1	0.06	1.00	no	-0.49	1.00	no
GAPDH	TC124579	Solyc07g005390.1	-0.41	1.00	no	0.82	1.00	no
Phosphoglycerate kinase	TC123837	Solyc07g066610.1	0.08	1.00	no	-0.14	1.00	no
Phosphoglycerate kinase	TC116028	Solyc07g066600.1	-0.18	1.00	no	-0.31	1.00	no
Catalase	TC115751	Solyc02g082760.2	-0.61	1.00	no	-1.33	1.00	no
Catalase	TC115865	Solyc12g094620.1	2.00	0.68	no	0.01	1.00	no
Cys protease	TC124125	Solyc08g082400.1	-0.34	1.00	no	0.64	1.00	no
Cys protease	TC116356	Solyc12g095910.1	2.19	0.26	no	-0.34	1.00	no
α-Tubulin	TC115716	Solyc04g077020.2	0.94	1.00	no	0.54	1.00	no
Ubiquitin	TC115896	Solyc07g064130.1	0.33	1.00	no	-1.05	1.00	no
Actin	TC124219	Solyc04g011500.2	-0.28	1.00	no	-0.54	1.00	no
DNAJ chaperone	TC124053	Solyc11g006460.1	0.52	1.00	no	-0.65	1.00	no
Translation initiation factor 5A	TC116211	Solyc12g010060.1	0.39	1.00	no	-0.43	1.00	no

^a^ Housekeeping genes are selected from reference [43] and converted TC accession into gene id by blast tool available in website of http://solgenomics.net/. R log_2_ fold change: log_2_ fold change of FPKM values after TYLCV infection/ FPKM values before TYLCV infection in resistant tomato variety. S log_2_ fold change: log_2_ fold change of FPKM values after TYLCV infection/ FPKM values before TYLCV infection in TYLCV susceptible tomato variety. The FPKM values in two tomato varieties before and after TYLCV infection are showed in Table S1. An absolute value of log_2_ fold change >1 and the False Discovery Rate (FDR) < 0.05 was set to declare differentially expressed genes.

In the comparison of gene expression levels before and after TYLCV infection, an absolute value of log_2_ fold change >1 and the False Discovery Rate (FDR) < 0.05 was set to declare differentially expressed genes (DEGs) involved in the response of tomato to TYLCV infection. It is notable that the defense responses of these two tomato lines were quite different, with 209 and 807 genes differentially expressed in the R and S lines, respectively (Table S2). However, the proportion of up-regulated DEGs was higher in the R line (58.37%) than in the S line (9.17%). Moreover, thirty-eight DEGs were common in both R and S lines and eleven (28.95%) common genes were up-regulated in the R line, whereas all of the thirty-eight common genes were down-regulated in the S line.

### Gene Ontology Analysis of Differentially Expressed Genes

**Figure 1:**
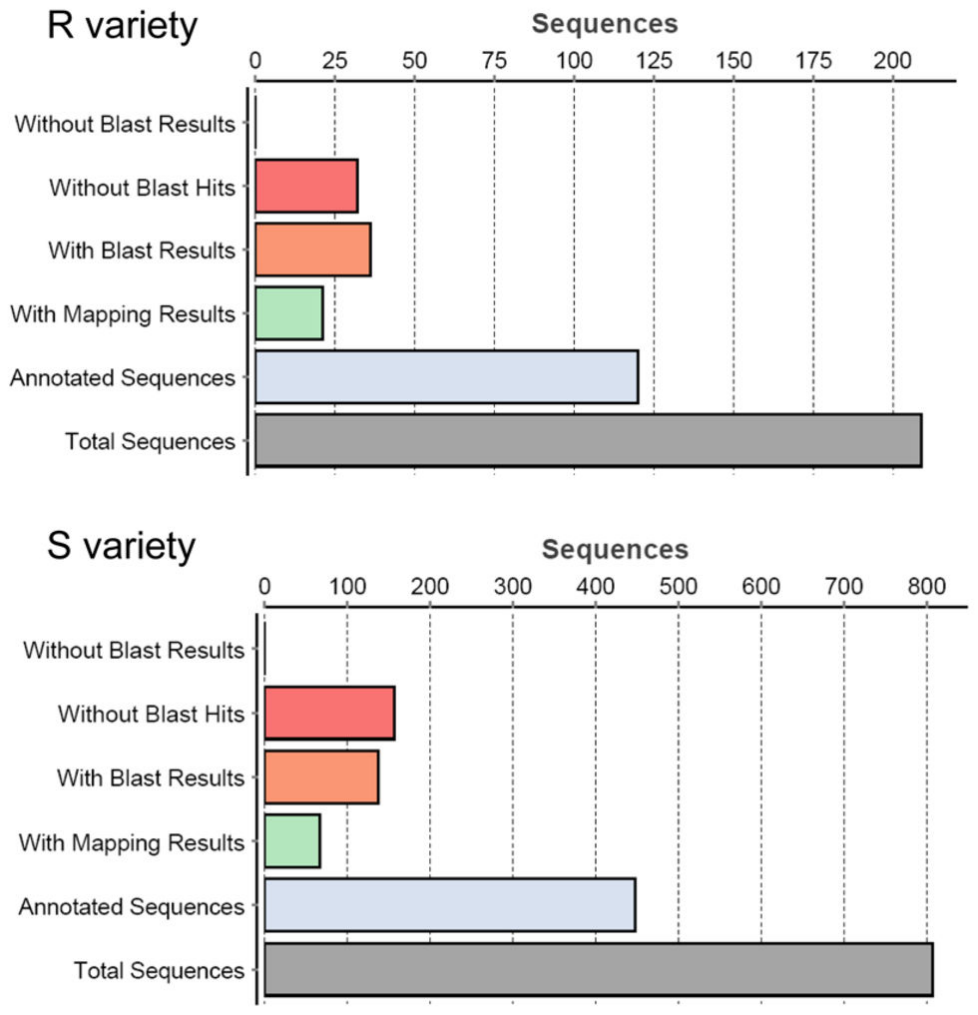
Annotation statistics of the defferentially expressed genes in the R and S lines. Annotation results were obtained from blast2go software with default parameters. R variety: TYLCV-resistant tomato breeding line CLN2777A, S variety: TYLCV-susceptible tomato breeding line TMXA48-4-0.

**Figure 2:**
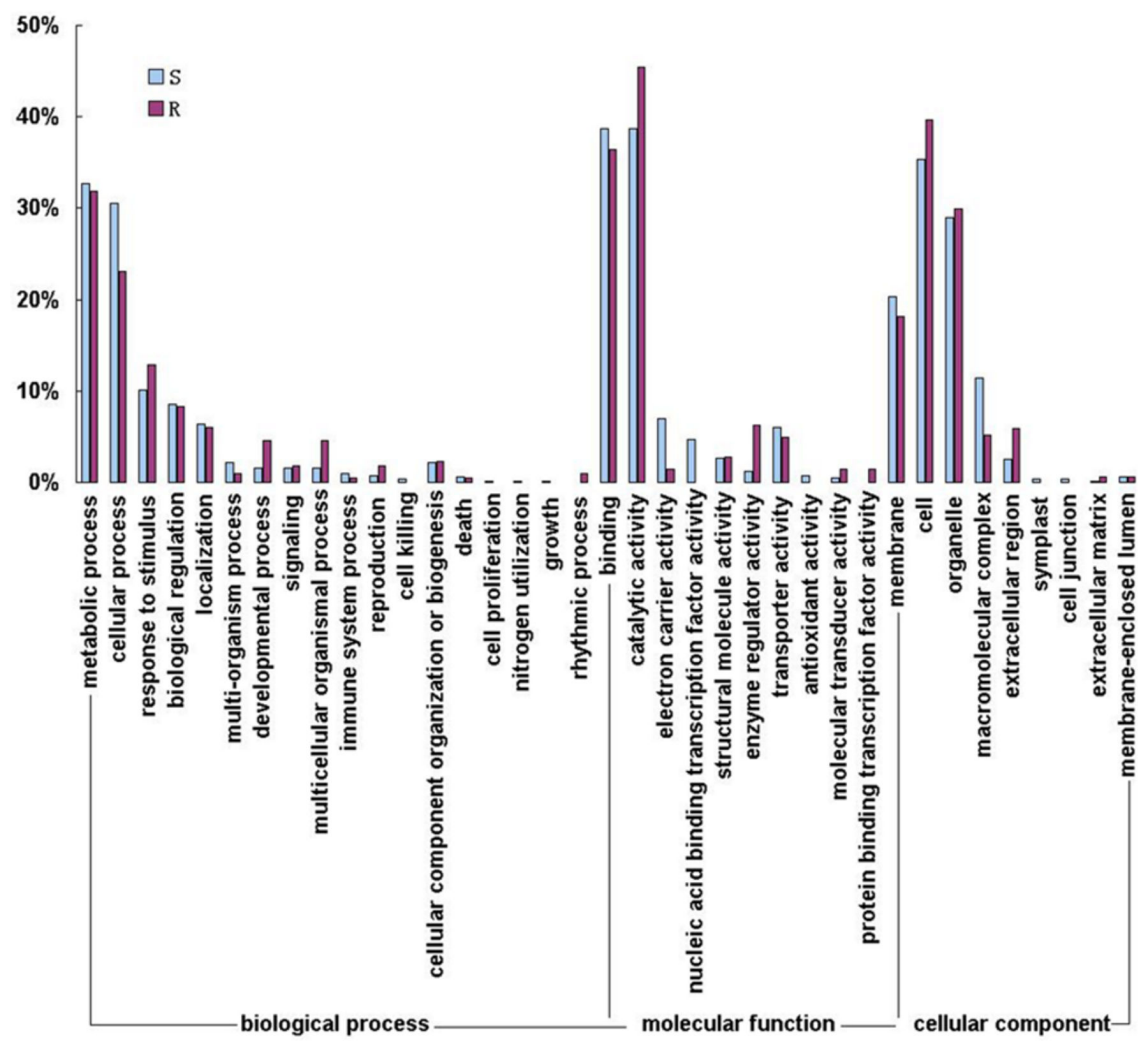
GO categories of differentially expressed genes in the R and S lines. R: TYLCV-resistant tomato breeding line CLN2777A, S: TYLCV-susceptible tomato breeding line TMXA48-4-0. The percent of DEGs which belong to three major functional categories (biological process, cellular component and molecular function) was shown.

For a better understanding of DEGs involved in the response of tomato to TYLCV infection, the functional classes of DEGs were subjected to gene ontology (GO) analysis with blast2go software. Blast2go software yielded annotated sequences for 67.46% and 63.69% of DEGs in the R and S lines, respectively (Figure 1). Within the biological process class, a large number of DEGs were placed in the categories of metabolic process, cellular process, response to stimulus, biological regulation and localization (Figure 2). The proportion of DEGs involved in the category of cellular processes was higher in the S line (30.50%) than that in the R line (23.04%), while the proportion of DEGs in the developmental processes, response to stimulus, and multicellular organismal processes was slightly higher in the R line than that in the S line. In addition, a few DEGs were found to specifically participate in cell killing, cell proliferation, nitrogen utilization and growth in the S line, while some DEGs were exclusively involved in the rhythmic process in the R line. Within the molecular function class, many DEGs showed binding activity, catalytic activity, transporter activity, structural molecule activity and enzyme activity. DEGs involved in the categories of nucleic acid binding transcription factor activity and antioxidant activity were specific to the S line By contrast, DEGs involved in protein binding transcription factor activity were found only in the R line. In the class of cellular components, most of the DEGs were found to be involved in proteins that are localized in the cell, organelle, membrane, macromolecular complex and extracellular region. Several DEGs were exclusively found in symplast and cell junction in the S line.

### Differential Expression of Two Tomato Lines in Response to TYLCV Infection

Dissection of expression levels of individual genes in the R and S lines revealed that many groups of genes were differentially modulated in response to TYLCV infection. WRKY transcription factors are one of the largest families of transcriptional regulators in plants and have been shown to modulate many plant processes including the process of pathogen associated molecular pattern (PAMP)-triggered immunity. Sixteen WRKY genes were identified as down-regulated DEGs in the S line with 3.84 ~ 7.89 fold changes. By contrast, the expression levels of seven WRKY genes were up-regulated in the R line (Table S3). 

Plants resistance genes (R genes) encode proteins that recognize avirulent proteins of pathogen and initiate plant defense mechanisms with a characteristic of hypersensitive response [44]. The expression of seven putative R genes including disease resistance RPP13-like gene (Solyc11g069670.1 and Solyc02g084890.1), TMV resistance gene (Solyc04g007320.1 and Solyc02g077060.1 ), *verticillium* wilt disease resistance Ve gene (Solyc09g005080) and disease resistance *Hcr2-0B* gene (Solyc06g008300.2) was significantly depressed in the S line after TYLCV infection, while they were either up-regulated or were unaffected by TYLCV infection in the R line (Table S4). 

Protein kinases and receptor (-like) kinases are well known in disease resistance either in a positive manner [45–48] or in a negative manner [49]. Thirty-two protein kinase genes and eight receptor (-like) kinase genes were identified as DEGs and 82.5% of them were down-regulated in the S line (Table S5). In contrast, only half of these kinase genes were slightly down-regulated and not identified as DEGs in the R line. 

### Putative Defense-Related Genes in Response to TYLCV Infection

Though various diversities between the R and S lines were revealed by the DEGs and GO analysis, we were particularly interested in those up-regulated DEGs in the R line. After going through references available, at least 40 annotated genes out of the 122 up-regulated DEGs in the R line were found to be associated with plant defense response at different levels, such as cell wall formation and reorganization, ethylene response, ubiquitination during plant immune signaling, metabolite synthesis, ranging from the regulation of transcription factors to the activation of defense genes and to the post-translational modification of proteins that participate in the defense response to pathogen infection (Table 3). For instance, one NAC transcription factor (Solyc05g007770.2) was up-regulated 7.83 folds and four ethylene response factor (ERF) genes (ERF4, DDTFR10/A, Solyc04g007000.1 and Solyc08g008300.1) were induced 4~ 6 folds in response to TYLCV infection, suggesting that they functioned in the regulation of disease resistance pathways. Two THT genes (THT7-1 and Solyc00g272810.1) were up-regulated in the R line compared to the S line. THT gene family encode the enzyme hydroxycinnamoyl-CoA:tyramine *N*-(hydroxycinnamoyl) transferase, which catalyzes the synthesis of two phenolic compounds, *p*-coumaroyloctopamine and *p*-coumaroylnoradrenaline. The elevated levels of *p*-coumaroyloctopamine and *p*-coumaroylnoradrenaline in tomato were accompanied by elevated mRNA levels of THT genes and were associated with the resistance-related response in tomato lines carrying the Cf*-9*, *Pto*, and *Fen* genes to the fungus *Cladosporium fulvum, Pseudomonas syringae* pathovar *tomato* and the organophosphorous insecticide fenitrothion, respectively [50]. The up-regulation of these two THT genes suggested the synthesis of defense-related phenolic compounds and their roles in TYLCV defense.

**Table 3:** A list of putative defense related genes from the up-regulated DEGs in the R line.

**Gene**	**Seq. Description**	**log_2_ fold change**	**Function and reference**
Solyc01g005470.2	protein plant cadmium resistance 2-like	3.74183	
Solyc07g006560.2	Hypersensitive response assisting protein	4.24441	
Solyc03g096840.2	hydrogen peroxide-induced 1	3.03148	
Solyc10g055780.1, Solyc10g055790.1	endochitinase 3-like	3.39235	[62]
ERF4	ethylene response factor 4	5.97883	[54–56]
DDTFR10/A	ethylene response factor 5	4.08545	[54–56]
Solyc08g008300.1	ethylene-responsive transcription factor erf061-like	4.697	[54–56]
Solyc04g007000.1	ap2 erf and b3 domain-containing transcription repressor tem1-like	6.7929	[54–56]
Solyc09g065780.2	3-ketoacyl- synthase 17-like	2.53957	Wax biosynthesis [63]
Solyc08g082640.2	cellulose synthase-like protein g3-like	4.37788	Cell wall formation [61]
Solyc01g111340.2, Solyc01g111350.2	probable endo-beta-xylanase c-like	3.23789	Cell wall reorganization [61]
LEXYL2	beta-xylosidase alpha-l-arabinofuranosidase 2-like	3.06397	Cell wall reorganization [61]
Solyc05g052670.1	bahd acyltransferase dcr-like	6.88485	Pathogen defense [64]
Solyc07g006570.2	aleurone ribonuclease	2.92916	Host defense [65]
CHRD	ribonuclease uk114-like	3.12484	Host defense [65]
PMEU1	pectin methylesterase	2.70772	Restrict fungal infection by *Botrytis cinerea* [66]
CYP-3	cysteine proteinase 3-like	3.61352	*Rcr3* is required for the function of Cf*-2* gene [67]
Solyc10g080610.1	f-box family protein	2.71334	Ubiquitination during plant immune signaling [52,68]
Solyc01g100000.2	f-box protein pp2-b15-like	4.36437	Ubiquitination during plant immune signaling [52,68]
Solyc01g079530.2	e3 ubiquitin-protein ligase march3	3.79656	Ubiquitination during plant immune signaling [52,68]
Solyc08g006770.2	flavonol synthase flavanone 3	4.68864	Soybean mosaic virus resistance [69]
Solyc03g043740.2	hydroxyproline-rich glycoprotein family protein	4.49604	Plant defense against pathogen infection [70]
LAPA1	leucine aminopeptidase	3.42183	Inducible defense component to *Pseudomonas syringae* [71]
Solyc00g187050.2	leucine aminopeptidase	3.22901	Inducible defense component to *Pseudomonas syringae* [71]
Solyc07g007250.2	metallocarboxypeptidase inhibitor precursor	3.41604	Up-regulation in wounded tomato leave [72]
Solyc05g007770.2	nac transcription factor 29-like	7.82817	Regulating jasmonic acid-signaled defense responses [73]
Solyc05g012580.1	nodulin-like protein	4.8075	Induced in leaves of *Medicago truncatula* in response to pathogenic bacteria [74]
Solyc02g076690.2	oryzain alpha chain-like	3.77976	Up-regulated in leaves of mycorrhizal rice plants [75]
Solyc08g080670.1	osmotin-like protein	6.85817	Blocking the growth of *Phomopsis viticola* and *Botrytis cinerea* mycelia [76]
Solyc09g011580.2	probable glutathione s-transferase-like	2.66912	[77]
Solyc07g008560.2	probable inactive purple acid phosphatase 27-like	4.18825	Basal resistance against *Pseudomonas syringae* [78]
Solyc01g010480.2	protein twin lov 1-like	3.58591	Defense response to *Cochliobolus victoriae* [79]
Solyc09g084480.2	proteinase inhibitor i	4.50463	Defense against oomycete pathogens [80]
Solyc09g084490.2	proteinase inhibitor i	2.57572	Defense against oomycete pathogens [80]
Solyc09g089510.2	proteinase inhibitor i-b-like	3.65828	Defense against oomycete pathogens [80]
Solyc02g090390.2	serine threonine-protein kinase srk2i-like	3.75423	Resistance to *Pseudomonas* [46]
Solyc07g055720.2	small heat-shock	6.20304	Specific restriction of tobacco Etch virus [81]
Solyc01g087800.2	subtilisin-like protease-like	4.54133	Induced by pathogen [82], regulator of primed immunity [83]
SBT4C	subtilisin-like protease-like	2.98735	Induced by pathogen [82], regulator of primed immunity [83]
TD	threonine deaminase	3.47734	Defenses against *Manduca sexta* [84]
Solyc06g083470.2	tropinone reductase homolog at1g07440-like	4.46584	Up-regulated in viral infected grapevines [85]
Solyc00g272810.1	tyramine n-feruloyltransferase 4 11-like	6.56522	Synthesis of phenolic compounds [50]
THT7-1	tyramine n-feruloyltransferase 4 11-like	3.2959	Synthesis of phenolic compounds [50]

### Quantitative RT-PCR Validation of RNA-seq Expression

**Figure 3:**
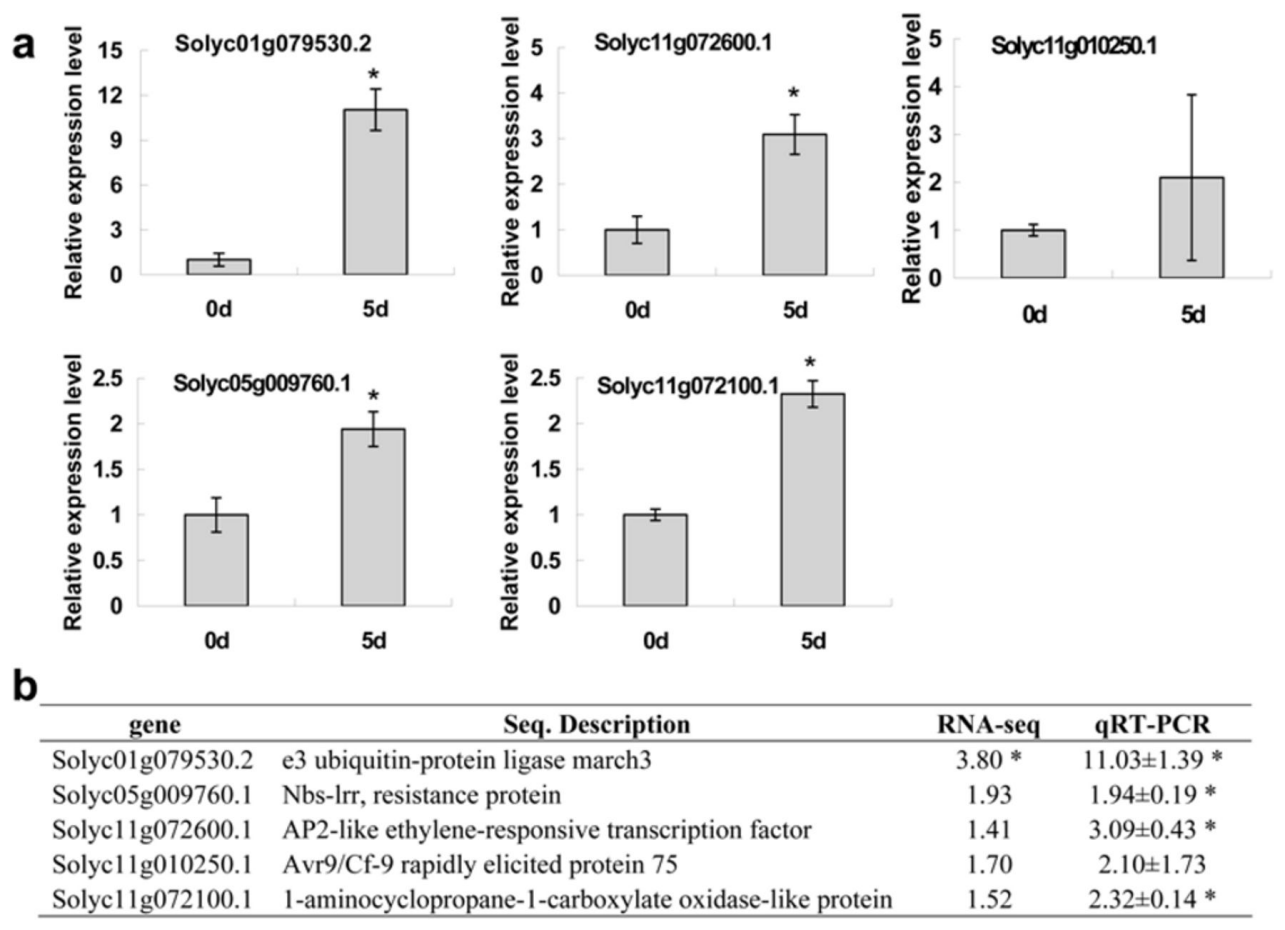
Validation of gene expression with quantitative RT-PCR. a: Quantitative RT-PCR was used to measure the relative expression levels of five pathogen resistance related genes in the R line, with the tomato α-Tubulin (Solyc04g077020.2) as an internal reference. Values were expressed as fold changes of transcript levels in the TYLCV inoculated leaf samples at 5 dpi with respect to the transcript levels in non-inoculated samples at 0 dpi. Error bars represented SE of three biological replicates and significant differences by Student’s *t* test for P< 0.05 are indicated by asterisks. b: Comparisons of transcripts fold changes as detected by RNA-seq and quantitative RT-PCR for the five pathogen resistance related genes in the R line. Asterisks indicated the differentially expressed gene revealed by RNA-seq or significant differences in quantitative RT-PCR analysis by *t* *test* (P= 0.05).

In order to validate the gene expression data from RNA-seq, the expression levels of five pathogen resistance related genes were determined by quantitative RT-PCR (primers were listed in Table S6) in the R line. With the exception of one resistance related gene (Solyc11g010250.1), four of these tested genes were characterized by a significant up-regulation in response to TYLCV infections at 5 dpi, though three of them were not counted as DEGs in RNA-seq analyses (Figure 3). Nonetheless, all tested genes revealed a similar trend of transcript accumulation as in RNA-seq analyses, indicating that changes in expression determined by RNA-seq were accurate. 

### VIGS Validation of Candidate Resistant Gene

**Figure 4:**
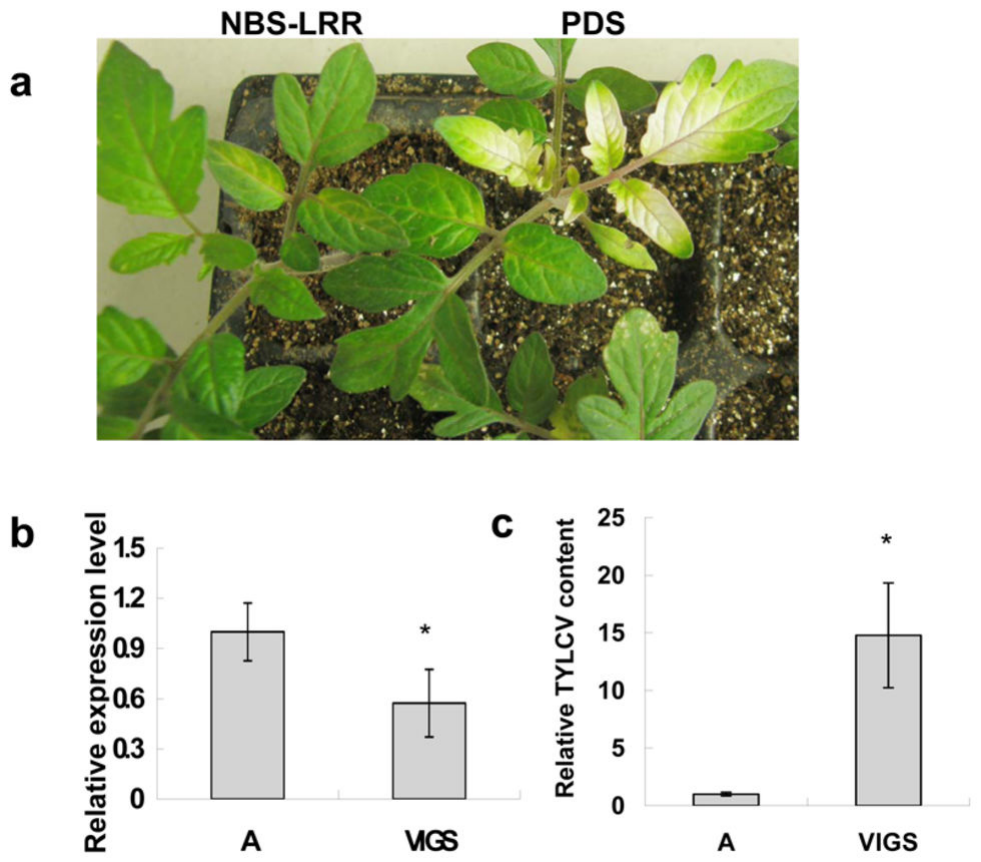
Validation of putative resistant gene with virus-induced gene silencing. a: Cotyledon agroinfiltration of TRV vectors was carried out in the R plantlets at the cotyledon stage. R plantlets treated with NBS-LRR resistant gene silencing constructs pTRV1 and pTRV2 - (NBS-LRR) showed normal phenotype (left), R plants treated with *Phytoene* *desaturase* (PDS) gene silencing constructs pTRV1 and pTRV2 -(PDS) showed bleached areas in leaflets (right, positive control). b: quantitative RT-PCR analyzed the relative expression levels of the NBS-LRR resistant gene in the VIGS-treated plants one month after agroinfiltration with TRV vectors. The tomato α-Tubulin (Solyc04g077020.2) was used as an internal reference. Error bars represented SE of three biological replicates and significant differences by Student’s *t* test for P< 0.05 are indicated by asterisks. “A” represented the R plantlets treated with pTRV1 and empty pTRV2 (negative control), “VIGS” represented the R plantlets treated with pTRV1 and pTRV2-(NBS-LRR). c: TYLCV accumulation in the NBS-LRR resistant gene silenced plants was estimated with total genomic DNA by quantitative RT-PCR. Values were normalized using the tomato α-Tubulin (Solyc04g077020.2) as an internal reference. Error bars represented SE of three biological replicates and significant differences by Student’s *t* test for P< 0.05 are indicated by asterisks.

To investigate the resistant role to TYLCV, a NBS-LRR resistance gene (Solyc05g009760.1) which was induced about 2- fold in the R line (Figure 3) was further challenged with TYLCV after VIGS at the cotyledon stage. One month after agroinfiltration, the success of TRV silencing system was confirmed by the appearance of bleached areas in the leaves of R plantlets treated with pTRV1 and pTRV2-PDS (Figure 4a), and the expression level of the NBS-LRR resistance gene after silencing was decreased 50% compared to the negative control (Figure 4b). Meanwhile, total genomic DNA of TYLCV-infected R plantlets was extracted for detection of virus accumulation. Quantitative RT-PCR revealed that the amount of virus accumulation in the VIGS-treated R plants was about 15 times more than the negative control when α-Tubulin was used as an internal reference (Figure 4c), but no disease symptoms of leaf curling and yellowing were observed in these VIGS-treated R plants.

## Discussion

Several studies utilizing proteomic, metabolomic and gene network have documented the global responses of tomato to TYLCV infection [14–20]. In this study, we extended the fundamental understanding of tomato response to TYLCV infection through comparing whole transcriptome changes between a TYLCV-resistant (R) line and a TYLCV-susceptible (S) line. We discovered that the defense response of these two tomato lines to TYLCV infection were quite different. First, we identified 209 DEGs in the R line wherein 58.37% DEGs were up-regulated in response to TYLCV infection. Conversely, among the 807 DEGs identified in the S line, only 9.17% were induced after TYLCV infection. Similar observations were found in the comparative transcriptome profiling between blast-resistant and blast-susceptible rice, which may be attributed to the successful establishment of infection in the susceptible variety [32]. Secondly, DEGs from the R and S lines shared 38 common genes; however, 11 (28.95%) of these common genes were up-regulated in the R line, while all of the 38 common genes were down-regulated in the S line. Thirdly, gene ontology analyses revealed that similar GO terms existed in the DEGs from both lines, but despite this commonality, some sets of defense related genes and their expression levels were dissimilar in these two tomato lines. Genes encoding WRKY transcriptional factors, R genes, protein kinases and receptor (-like) kinases exhibited a dramatic down-regulation in the S line. These genes are known to have a link to defense responses in plants [44,51–53]. The greater extent of down-regulation of these defense related genes is likely related to the fast infection progression of TYLCV in the S line. In contrast, most of these gene sets were identified as up-regulated or not differentially expressed in the R line. These different reactions may be attributed to the successful establishment of resistance to TYLCV in the R line, and the susceptible response in the S line.

Assuming that genes involved in TYLCV resistance would be expressed at higher levels in TYLCV resistant tomato vs TYLCV susceptible tomato, the group of Henryk Czosnek attempted to compare the trancriptomes of two inbred tomato lines generated from the same breeding program before and after inoculation, however, they did not obtain any significant results ([17], and references therein). The nearly same genetic background of these two inbred tomato lines may contribute to the difficulty in distinguishing of differentially expressed genes. One purpose of our study was to identify genes critically involved in early response to TYLCV infection, and we prepared pooled cDNA libraries from samples at 3, 5 and 7 dpi, according to the cDNA library preparation strategy for screening preferentially expressed genes in TYLCV infected tomato which could represent gene expression at the early stages of infection and avoid the secondary effects of infection-related cell damage that may occur with the appearance of symptoms [17]. With two tomato varieties carrying different genetic backgrounds, we identified 122 up-regulated DEGs specific in the R line compared to the S line, and at least 40 annotated up-regulated DEGs such as genes encoding hypersensitive response assisting protein, ethylene response factor [54–56], serine threonine-protein kinase [45–48], NAC transcription factor [57], f-box protein [52], glutathione s-transferas [58], glycosyltransferase/glucosyltransferase [59,60], cellulose synthase-like protein [61] and so on, were found to be associated with tomato defense response at different levels (Table 3). These up-regulated DEGs may function ranging from the regulation of transcription factors to the activation of defense genes and to the post-translational modification of proteins that participate in the defense response to pathogen infection. Comparison of the up-regulated DEGs in the R line revealed a little overlap with those 69 genes preferentially expressed in TYLCV resistant tomato identified by screening cDNA libraries from infected and uninfected TYLCV resistant and TYLCV susceptible tomatoes [17], with the only one gene (Solyc04g071140.2) coding a decarboxylase protein showed the same up-regulation trend. This can be ascribed to different genotypes and genetic backgrounds of the tomato varieties under investigation. However, not all the genes preferentially expressed in resistant plants play the same role in the establishment of resistance to TYLCV [18]. Twenty-five preferentially expressed genes in TYLCV resistance tomato were silenced by TRV-mediated silencing system, but only 5 genes led to the collapse of resistance in tomato [17–20]. Moreover, investigation of the transcripts, which did not show altered expression level, but had high log_2_ fold changes (like more than 2.5 log_2_ fold change in response to cytokinins [34]), could provide more information for gene function dissection. Thus, in order to identify genes conferring TYLCV resistance in the R line, further validation of the 40 up-regulated DEGs as well as resistance related non-DEGs with high log_2_ fold changes in our comparative transcriptome should be determined by quantitative RT-PCR. In our quantitative RT-PCR experiments, four genes involved in pathogen resistance in plants were induced significantly with consistent trends of expression levels revealed in RNA-Seq analyses (Figure 3), although three of them were not identified as differentially expressed genes in RNA-seq analyses. 

Like other plants, tomato resistance to TYLCV may involve in a complicated gene network, which may start with a basal response and production of general pathogen-associated molecular pattern molecules (PAMPs), followed by activation of the MAPK-signaling cascades and production of antimicrobial compounds, and finally the genes specific for response to TYLCV are expressed [17–20]. The transcriptome here provided valuable data for further characterization of candidate genes that are responsive to TYLCV infection and are involved in the resistant gene network. Indeed, silencing of one gene, which was not identified as DEG in this study facilitated the accumulation of TYLCV in the VIGS-treated R tomato plants when comparing to the wildtype (Figure 4). This also indicated that VIGS is an exquisite and quick means to identify genes resistant to TYLCV infection, and it provides comprehensive knowledge about the molecular mechanisms underlying the resistance gene(s) network.

## Supporting Information

Table S1 **Lists of mapped transcripts in CLN2777A and TMXA48-4-0.** FPKM b: the FPKM values before TYLCV infection. FPKM a: the FPKM values after TYLCV infection. Log_2_ fold change= log_2_ (FPKM a / FPKM b). An absolute value of log_2_ fold change >1 and the False Discovery Rate (FDR) < 0.05 was set to declare differentially expressed genes. Genes without names were novel transcripts.
(XLS)

Table S2 **Lists of differentially expressed genes in CLN2777A and TMXA48-4-0.** FPKM b: the FPKM values before TYLCV infection. FPKM a: the FPKM values after TYLCV infection. Log_2_ fold change= log_2_ (FPKM a / FPKM b). An absolute value of log_2_ fold change >1 and the False Discovery Rate (FDR) < 0.05 was set to declare differentially expressed genes. Genes without names were novel transcripts.
(XLS)

Table S3 **Differential expression of sixteen WRKY genes in and TMXA48-4-0.** FPKM b: the FPKM values before TYLCV infection. FPKM a: the FPKM values after TYLCV infection. Log_2_ fold change= log_2_ (FPKM a / FPKM b). An absolute value of log_2_ fold change >1 and the False Discovery Rate (FDR) < 0.05 was set to declare differentially expressed genes.
(XLS)

Table S4 **Differential expression of seven R genes in CLN2777A and TMXA48-4-0.** FPKM b: the FPKM values before TYLCV infection. FPKM a: the FPKM values after TYLCV infection. Log_2_ fold change= log_2_ (FPKM a / FPKM b). An absolute value of log_2_ fold change >1 and the False Discovery Rate (FDR) < 0.05 was set to declare differentially expressed genes.
(XLS)

Table S5 **Differential expression of forty kinase genes in CLN2777A and TMXA48-4-0.** FPKM b: the FPKM values before TYLCV infection. FPKM a: the FPKM values after TYLCV infection. Log_2_ fold change= log_2_ (FPKM a / FPKM b). An absolute value of log_2_ fold change >1 and the False Discovery Rate (FDR) < 0.05 was set to declare differentially expressed genes.
(XLS)

Table S6 **Primers of selected genes for quantitative RT-PCR.**
(DOC)
