# Supplementary material for: Comparative Transcriptome Profiling of a Resistant vs. Susceptible Tomato (Solanum lycopersicum) Cultivar in Response to Infection by Tomato Yellow Leaf Curl Virus
Source: PLoS One. 2013 Nov 18;8(11):e80816. doi: 10.1371/journal.pone.0080816 (PMC3832472; doi:10.1371/journal.pone.0080816)
Supplement: Table S6 — Primers of selected genes for quantitative RT-PCR. (DOC) [file pone.0080816.s006.doc]

| Gene | **Primers (5’→ 3’)** | **Seq. Description** |
| --- | --- | --- |
| Solyc01g079530.2 | GAAAGAGAAATGCTGGTGAGGAGT | e3 ubiquitin-protein ligase march3 |
|  | CTCCTCCATTCATCACCTCTAACA |
| Solyc05g009760.1 | ACTATTATTCTCCCCTCGCGTCTC | Nbs-lrr, resistance protein |
|  | GTAAGCACCGAGAACCCAATGACT |
| Solyc11g072600.1 | CCGCAATCAAATGTAATGGAAG | AP2-like ethylene-responsive transcription factor |
|  | AACTATCGGTGAACCAGATTTGTC |
| Solyc11g010250.1 | AGGAGGGGGGTTGAAGAAGAGTA | Avr9/Cf-9 rapidly elicited protein 75 |
|  | AAACCCTTCTTTCTTCTTTTTTCCA |
| Solyc11g072100.1 | ATGAGCAACGATAGGTACAAGAGC | 1-aminocyclopropane-1-carboxylate oxidase-like protein |
|  | TGGGCACTGACACTCTGTTTCTAC |
| Solyc04g077020.2 | TGACGAAGTCAGGACAGGAA | α-Tubulin |
|  | CTGCATCTTCTTTGCCACTG |
